# Antiplasmodial activity of *Cocos nucifera* leaves in *Plasmodium berghei*-infected mice

**DOI:** 10.1007/s12639-020-01207-7

**Published:** 2020-03-09

**Authors:** Nicole M. Tayler, Rosa De Jesús, Rita Spadafora, Lorena M. Coronado, Carmenza Spadafora

**Affiliations:** 1 Centro de Biología Celular y Molecular de Enfermedades (CBCMe), Instituto de Investigaciones Científicas y Servicios de Alta Tecnología, City of Knowledge, Bldg. 208, P.O. Box 0843-01103, Panama, Republic of Panama; 2 Department of Biotechnology, Acharya Nagarjuna University, Guntur, India; 3 Asociación Panameña para la Conservación de la Naturaleza (ANCON), Panama, Republic of Panama

**Keywords:** *Plasmodium falciparum*, *Cocos nucifera*, Aqueous extract, Antiplasmodial, *Plasmodium berghei*

## Abstract

*Plasmodium falciparum* (*P. falciparum*) malaria presents serious public health problems worldwide. The parasite´s resistance to antimalarial drugs has proven to be a significant hurdle in the search for effective treatments against the disease. For this reason, the study of natural products to find new antimalarials remains a crucial step in the fight against malaria. In this study, we aimed to study the in vivo performance of the decoction of *C. nucifera* leaves in *P. berghei*-infected mice. We analyzed the effectiveness of different routes of administration and the acute toxicity of the extract. Additionally, we determined the suppressive, curative and prophylactic activity of the extract. The results showed that the decoction of leaves of *C. nucifera* is most effective when administered intramuscularly to mice in comparison to intraperitoneal, subcutaneous and intragastric methods. We also found that organ signs of acute toxicity appear at 2000 mg/kg/day as evidenced by necropsy examination. Additionally, we found that the prophylactic effect of the extract is of 48% inhibition, however, there is no curative effect. Finally, in a 4-day suppressive assay, we found that the extract can inhibit the growth of the parasite by up to 54% at sub-toxic doses when administered intramuscularly.

## Introduction

The most dangerous form of malaria is caused by *Plasmodium falciparum* (*P. falciparum*) parasites which present serious health problems worldwide. For the year 2017, there were approximately 219 million cases of malaria worldwide, compared to 217 million cases in the previous year. There were approximately 435,000 deaths from malaria globally in 2017, compared to 451,000 estimated deaths in 2016. The highest disease burden is centered in Sub-Saharan Africa, with children less than 5 years of age being the most affected group (WHO 2018). To date, the most effective ways to control these parasitic illnesses have been the use of drugs to treat the diseases and insecticides to control their transmission. Resistance to chloroquine, and lately to artemisinin derivatives and insecticides, gives fresh impetus to the search for new agents (Achan et al. 2018).

This search has led scientists to examine microorganisms, animal fur and plants (Higginbotham et al. 2014; Martínez-Luis et al. 2012; Durant et al. 2014: Isaka et al. 2018; Schorderet Weber et al. 2019; Whitman et al. 2019) for signs of activity against various diseases. In Panama we can find one of the planet’s most uniquely biodiverse environments, rich in plant and animal species potentially useful for alternative medicine studies. As part of their conservation efforts, The National Association for the Conservation of Nature (ANCON) protects a private natural reserve in Punta Patiño, Darién. This area is characterized by large extensions of coconut palm plantations of *Cocos nucifera* (*C. nucifera*).

The coconut tree has traditionally been used to treat several human diseases such as arthritis and asthma; and symptoms such as diarrhea and fevers; as a diuretic, and to treat malaria, among others (Lima et al. 2015). Several studies have described the traditional use of *C. nucifera* husk, water and inflorescence on mice and rats to address several pathologies (Naskar et al. 2011; Rinaldi et al. 2009; Renjith et al. 2013; Alviano et al. 2004; Omoboyowa et al. 2016). One study describes the use of *C. nucifera* leaf extracts as reducing amyloid-β_(1-42)_ aggregation in transgenic *Caenorhabditis elegans* (Manalo et al. 2017). Another study describes the safety of *C. nucifera* leaf extracts in mice, however, it does not evaluate the effectiveness of these extracts on any pathology (Paul et al. 2012). Because we have previously reported the in vitro antiplasmodial activity of the water extract of leaves (Tayler et al. 2019), and because to our knowledge there are no rigorous studies which describe the use of the leaves of *C. nucifera* against *P. falciparum*, we decided to test its in vivo potential as an antimalarial.

Our work focused on evaluating the inoculation route, acute toxicity, hematological parameters and antiplasmodial activity exerted by the aqueous extract of *C. nucifera* leaves from the Punta Patiño Private Natural Reserve in mice infected with *Plasmodium berghei* parasites.

## Materials and methods

### Ethics statement

All experimental procedures were performed in accordance with the institutional guidelines from the Institutional Committee of Animal Use and Care (Comité Institucional de Cuidado y Uso de Animales-CICUA) under approval number CICUA-17-004.

The authors were permitted to collect *C. nucifera* samples by the owners of the reserve, the National Association for the Conservation of Nature (ANCON), a non-government organization and stakeholder of this study. No other permission was required for research purposes and the field studies did not involve any endangered species.

### Plant material

Leaves of *C. nucifera* were collected from the Punta Patiño Natural Reserve in Darien, Panama. They were identified by a botanist and voucher samples of the leaves were deposited at the Herbarium of the Universidad de Panama with registry number 0111279. The extracts were prepared as described in previous work by Tayler et al. (Tayler et al. 2019). In brief, 15 g of leaves were cleansed using distilled water and delicate task wipes (Kimwipes™, Thermo Fisher Scientific, Waltham, MA, USA). After drying, leaves were cut into smaller pieces and macerated with a mortar and pistil. The leaves were placed into a sterile beaker, covered with 250 mL of distilled water, and boiled for 15 min. Afterwards, leaves were subsequently filtered through sterile 0.8 µm and 0.22 µm membranes (Nalgene^®^ vacuum filtration system). To prepare the experimental samples, 250 µL of the extract were aliquoted into glass vials and concentrated to reach approximately 292 mg/mL. Samples were aliquoted and stored at 4 °C until use. As positive control in all tests, chloroquine (10 mg/Kg/day) was applied subcutaneously.

### Mice and parasites

The tests used throughout these studies were carried out on female mice of the inbred line C57BL/6, of 7 weeks of age with average weights of 21.5 ± 0.5 g, produced and maintained in the animal facility of INDICASAT AIP at a temperature of 20 °C, with light cycles of 12 h light/12 h darkness. Mice were maintained under biological barrier conditions such as change of personal wardrobe, use of masks and gloves and sterilization of the bedding chips and food (121 °C/15 min). Mice were handled according to the regulations on the use of laboratory animals of INDICASAT AIP. The parasites used to produce the infection were *P. berghei* ANKA (chloroquine sensitive) which were maintained in mice by successive C57BL/6 mouse - mouse passages.

### Determination of route of administration

Female mice of the consanguineous line C57BL/6 were inoculated through intraperitoneal injection with 200 µl of *P. berghei* parasites. Four days after inoculation, infected blood was extracted through cardiac puncture and used to inoculate test subjects. A total of 10 mice were divided into 5 groups of two mice each, which were identified by permanent tattooing. These groups were characterized as: intramuscular (IM) administration, subcutaneous administration (SC), intragastrical (IG) administration, untreated group (control) and a chloroquine treated group injected subcutaneously with a 10 mg/kg dose (positive control) (Sigma, St. Louis, Missouri, USA). Mice were inoculated intraperitoneally with approximately 1 × 10^7^ of *P. berghei* parasites (Alger 2001) and treatment started 24 h later. The extract was administered at a dose of 100 mg/kg/day to each group using a 1 cc syringe (Nipro Medical Corporation, Bridgewater, NJ, USA) with a puncture at the respective site (IM and SC). Intragastric administration was performed using an oral gavage needle. The parasitemia was monitored until no more parasites were observed in the chloroquine group. To examine the parasitemia, a cut of approximately 1 mm was made on the tail of each mouse and blood smears were obtained which were subsequently stained with Giemsa 1:10, and parasites counted under a light microscope (brand OLYMPUS model BH2), with an objective of 100×.

### Toxicity assays

For the toxicity analysis, the OECD guideline on its acute toxic class method section for the testing of chemicals was followed (OECD 2002). After testing the suggested 2000 mg/kg/day dose, lower doses of 1000 and 750 mg/kg/day were also analyzed. Three experimental groups of 5 mice each were formed, with each of the concentrations above. A control group was treated with only the vehicle, which was sterile water. The chloroquine control group was treated with 10 mg/kg/day of drug. Another control group was that of untreated mice. The extract was administered via IM for 4 days for each of the concentrations. Animals were observed for gross signs of toxicity (lacrimation, hair erection and reduction in their motor and feeding activities) 4 h after the administration of the extract, then daily for 16 days to assess safety of the extract. All animals were retro-orbitally sampled for hemoglobin, hematocrit, mean corpuscular volume and white cell counts in three moments: prior to administration of the extract, 8 and 16 days after administration. The blood work was performed by the Laboratory of the School of Veterinary Science, University of Panama, Panama. Results are average values per groups (controls and treated) for each dose at day 1 (beginning of treatment), day 8 and day 16 (end of the experiment). The mice were weighed daily for 8 days and one final time 16 days after administration of the extract. At this point, the animals were sacrificed by overdose of the ketamine/xylazine anesthetic mixture (normal dose: 50-150/5-15 mg/kg body weight).

### 4-Day suppressive test

For the suppressive test, we followed the protocol by Al-Adhroey et al. (2011) with some modifications. This experiment was conducted on different days for each concentration, wherein each experimental group had separate control groups. For this, six groups of five (5) mice each were formed, of which two were the experimental groups: 500 and 750 mg/kg/day. Four groups formed the control groups, of which two were the chloroquine-treated mice and two were the negative control mice to which only the vehicle, sterile water, was added IM. Mice were inoculated intraperitoneally with about 1 × 10^7^*P. berghei* parasites and, 4 h post infection, treatment was started via IM and continued once a day for the next 4 days. Mean survival time (days) was evaluated over a 16-day period. Blood smears were obtained which were subsequently stained with Giemsa 1:10 and the number of parasites was counted under a light microscope (OLYMPUS model BH2), with an objective of 100×.

### Curative test

For the curative test, we followed the protocol by Al-Adhroey et al. (2011) with some modifications. Five groups of five mice each were formed; two groups were experimental, with 500 and 750 mg/kg/day of the extract and three control groups: one with only the vehicle (sterile water) all injected intramuscularly, one untreated and a group treated with subcutaneous chloroquine. Mice were inoculated with approximately 1 × 10^7^*P. berghei* parasites and 72 h post infection, treatment was started (D1) via IM and continued for an additional 5 days (D5). Blood smears were obtained daily, stained with Giemsa 1:10 and infected parasites were counted under a light microscope, with an objective of 100×. The antimalarial activities of the extract were compared to both control groups treated with only the vehicle and the reference groups treated with the standard drug (chloroquine). Mean survival time (days) was evaluated over a 16-day period.

### Prophylactic test

Again, following basically the protocol of Al-Adhroey et al. (2011), five groups of five mice each were formed; two groups were experimental, with 500 and 750 mg/kg/day of the extract and three control groups, one with only the vehicle, another of untreated animals and a chloroquine-treated group. All mice were given the extract IM for 72 h. On day 1 (D1), animals were inoculated with approximately 1 × 10^7^*P. berghei* parasites intraperitoneally and the treatment with the extract was continued for an additional 3 days (4 days total). The antimalarial activities of the extract were compared to control groups treated with only sterile water. Parasitemia was analyzed using Hoechst 33342 staining and flow cytometry analysis gated on the region of erythrocyte population as explained in another section below.

For all assays, chemosuppression was calculated with the following formula (Angeles et al. 2005):

$$Supression = \left( {\frac{Untreated group mean parasitemia - Treated group mean parasitemia}{Untreated mean parasitemia} } \right) \times 100$$

### Quantification of parasitemia using flow cytometry

Parasitemia was obtained as described in Tayler et al. (2019). Briefly, blood samples were treated with Hoechst 33342 (Invitrogen, USA) at 2 µg/mL and incubated for 30 min while protected from light. Additionally, background staining of an uninfected red blood sample was always performed (Spadafora et al. 2010) and used to define the population of red blood cells using FSC/SSC to be gated for all samples. Debris and other stained cellular populations were ungated. Acquisition was performed with a UV laser on a PARTEC CyFlow Space cytometer (Görlitz, Germany).

### Statistics

All data was analyzed using the software GraphPad (Prism, California, USA), performing analyses of variance. Significant differences between groups were determined using Bonferroni’s test. A p ≤ 0.05 was considered significant.

## Results

### Determination of route of administration

**Fig. 1:**
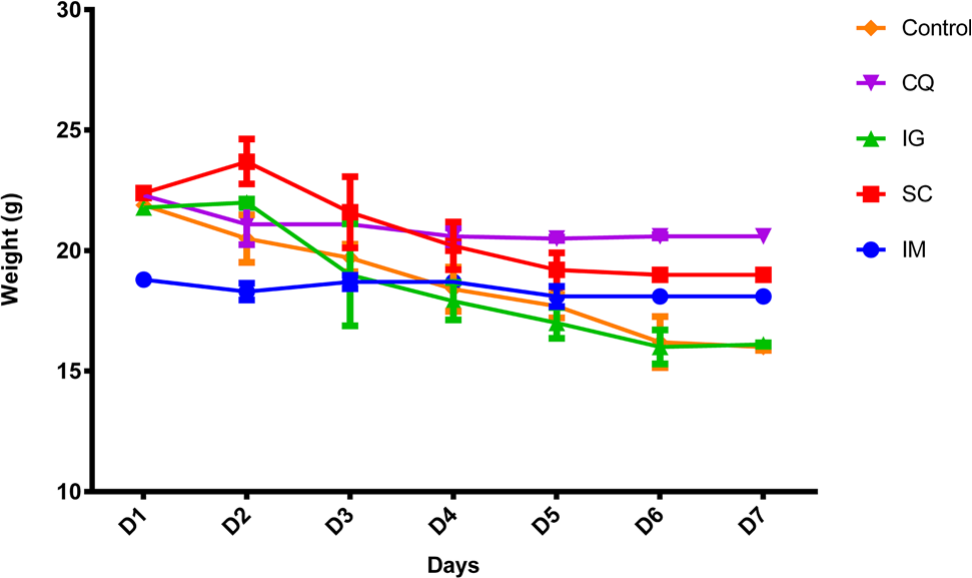
Weight of *P. berghei* infected mice by different inoculation routes. The graph represents the mean of two mice in each of the groups. IM, intramuscular; SC, subcutaneous; IG, intragastric; CQ, Chloroquine and control (untreated)

The results of testing different routes of administration of the extract determined that the most effective route was intramuscular (IM), since even though the weight of the mice decreased for all of the different treatments, the mice treated intramuscularly with the extract were the ones whose weight decreased the least (− 0.7 g), as shown in Fig. 1. With respect to parasitemia, mice given extracts via IM also had the lowest percentage of parasitemia (1.35%) at day 7, of all the extract-treated groups (Table 1).

**Table 1:** Parasitemia of *Plasmodium berghei* infected mice according to route of administration

Inoculation routes					
Days	IM	SC	IG	CQ	UT
1	0.10 ± 0.00	0.47 ± 0.0	0.40 ± 0.00	0.47 ± 0.0	0.60 ± 0.00
2	0.10 ± 0.07	1.67 ± 0.85	1.6 ± 0.85	0.03 ± 0.31	2.29 ± 1.20
3	0.00 ± 0.00	7.30 ± 3.98	8.6 ± 4.95	0.10 ± 0.05	9.80 ± 5.31
4	0.10 ± 0.07	8.95 ± 1.17	9.3 ± 0.49	0.10 ± 0.00	12.00 ± 1.56
5	0.10 ± 0.00	4.54 ± 3.12	16.00 ± 4.74	0.00 ± 0.07	15.00 ± 2.12
6	0.80 ± 0.49	6.10 ± 1.10	22.00 ± 4.24	0.00 ± 0.00	21.00 ± 4.24
7	1.35 ± 0.39	7.91 ± 1.28	37.00 ± 10.61	0.00 ± 0.00	36.00 ± 10.61

### Toxicity assays

In the group of mice given the concentration of 2000 mg/kg/day, 2 of the 5 mice died; which prompted the testing of two other lower dosages for acute toxicity (1000 and 750 mg/kg/day). In these other two groups no mice died and they survived until day 16 on which euthanasia was performed. In relation to weight, it was observed that no significant weight was lost relative to the controls at any of the three concentrations tested. With regard to the blood parameters, mice given the extract at 2000 mg/kg of weight had lower hematocrit and hemoglobin values than controls throughout the experiment. Mice given the extract at 1000 and 750 mg/kg of weight had normal values until the 8th day of treatment; however, all values for hematocrit and hemoglobin were lower than controls at day 16. All mice challenged with every concentration tested had values within the normal range of mean corpuscular volume, except for those treated with 750 mg/kg/day on Day 16 (VCM) (Table 2).

**Table 2:** Blood test values of uninfected mice treated with the decoction of *Cocos nucifera* leaves at day 1, day 8, and day 16 post treatment. Normal values are placed below each parameter title

Parameter	Day 1					
	2000		1000		750	
	Control	Treated	Control	Treated	Control	Treated
Hematocrit (%)	31.9 ± 1.22	35.32 ± 2.25	31.5 ± 1.70	34.54 ± 2.62	28.57 ± 6.54	28.76 ± 3.37
33–48%						
Hemoglobin (g/dL)	9.73 ± 1.7	11.66 ± 0.86	10.3 ± 0.35	11.5 ± 0.86	9.47 ± 2.16	9.58 ± 1.16
10–16						
MCV (fl/red cell)	50.23 ± 2.03	48.26 ± 2.3	48.33 ± 2.5	48.72 ± 1.33	50.23 ± 2.03	52.24 ± 2.94
42.3–55.2						

Parameter	Day 8					
	2000		1000		750	
	Control	Treated	Control	Treated	Control	Treated
Hematocrit (%)	32.2 ± 0.35	17.75 ± 15.54	26.4 ± 5.86	28.54 ± 4.99	23.33 ± 0.58	20.74 ± 8.88
33–48%						
Hemoglobin (g/dL)	10.67 ± 0.12	5.90 ± 5.16	8.77 ± 1.98	9.48 ± 1.69	7.73 ± 0.23	6.88 ± 2.96
10–16						
MCV (fl/red cell)	51.10 ± 0.40	61.15 ± 13.61	47.7 ± 3.42	50.24 ± 3.70	50.63 ± 1.70	51.52 ± 1.5
42.3–55.2						

Parameter	Day 16					
	2000		1000		750	
	Control	Treated	Control	Treated	Control	Treated
Hematocrit (%)	32.7 ± 0.85	33.25 ± 2.47	26.4 ± 5.86	28.54 ± 4.99	32.2 ± 0.35	21.96 ± 5.03
33–48%						
Hemoglobin (g/dL)	9.85 ± 1.34	11.05 ± 0.78	8.77 ± 1.98	9.48 ± 1.69	10.67 ± 0.12	7.28 ± 1.68
10–16						
MCV (fl/red cell)	50 ± 1.84	53.2 ± 1.84	47.7 ± 3.42	50.24 ± 3.7	51.1 ± 0.4	39.10 ± 12.2
42.3–55.2						

### 4-Day suppressive test

**Fig. 2:**
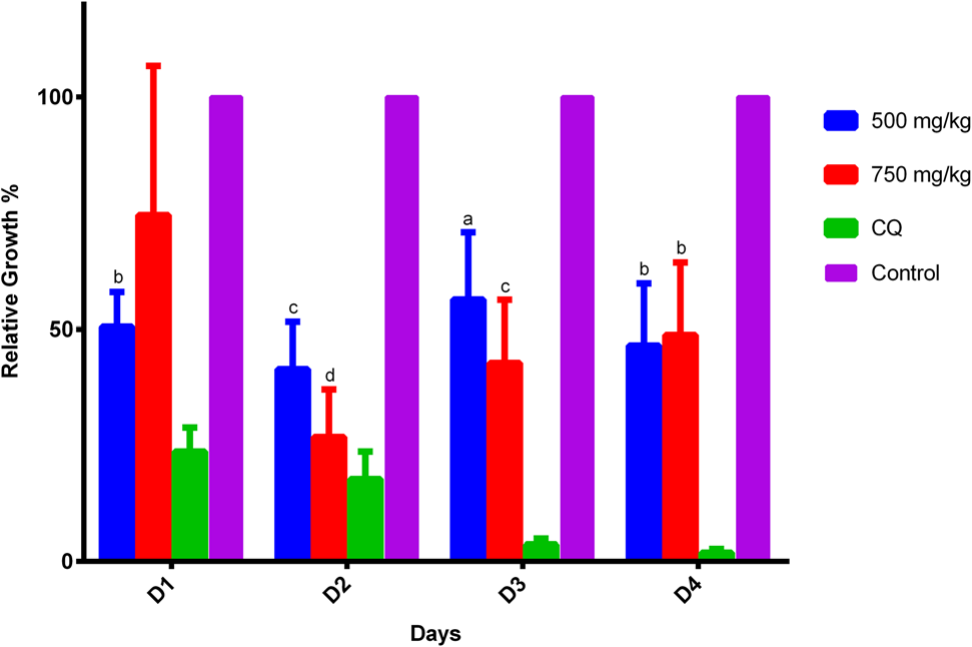
Treatment of infected mice with 500 and 750 mg/kg/day of the extract. Results shown are the mean of 5 mice in each group. Parasite growth was determined by microscopy and compared to controls with only vehicle added. Results shown are the mean of 5 mice ± SEM values. A p ≤ 0.05 was considered significant. a = p<0.05, b = p<0.01, c = p<0.005, d = p<0.0001

For the trials related to the 4-day suppressive test, treatments with 500 and 750 mg/kg/day of the extract were analyzed. The mean parasitemia on day 4 after infection was 11.8% ± 8.5 for 500 mg/kg/day and 7.4% ± 4.5 for 750 mg/kg/day. The range value for the control was 24.0 ± 1.2 to 16.1 ± 2.0. The percentage chemosuppression of the parasites was 51% for 500 mg/kg/day and 54% for 750 mg/kg/day. The daily suppressive effect can be observed in Fig. 2. Moreover, the mice infected with *P. berghei* parasites exhibited no signs of distress, such as weight loss, diminished exploratory behavior, hunched posture or unresponsiveness during the full length of the assay.

### Curative test

**Fig. 3:**
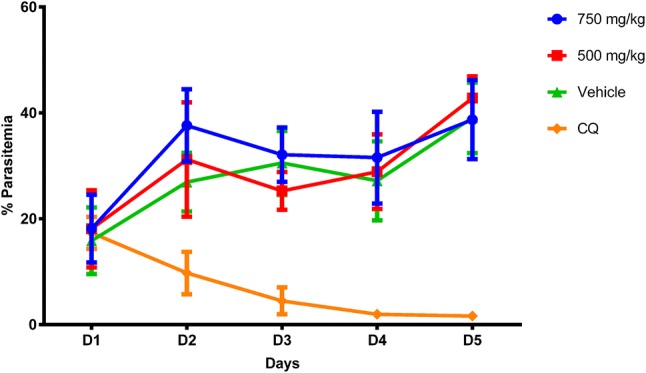
Curative assay of *C. nucifera* extract against *P. berghei*. Results shown are the mean of five mice ± SEM values. Parasite growth was determined by microscopy and compared to culture controls with only vehicle added. Day 1 [D1] is the 1 day of treatment after 72 h of untreated parasite infection

The parasitemia percentage on day 5 in mice infected and treated with 500 and 750 mg/kg/day was 42.80 ± 4.10 and 38.73 ± 7.46, respectively, while the untreated group had a percentage parasitemia of 39.08 ± 6.65, and that of the chloroquine-treated group was 1.64 ± 0.23. From the point of view of chemosuppression of the parasites, the mice treated with 500 and 750 mg/kg/day presented an inhibition of − 9.51% and 0.91%, whereas those treated with chloroquine had 95.81% (Fig. 3).

### Prophylactic test

**Fig. 4:**
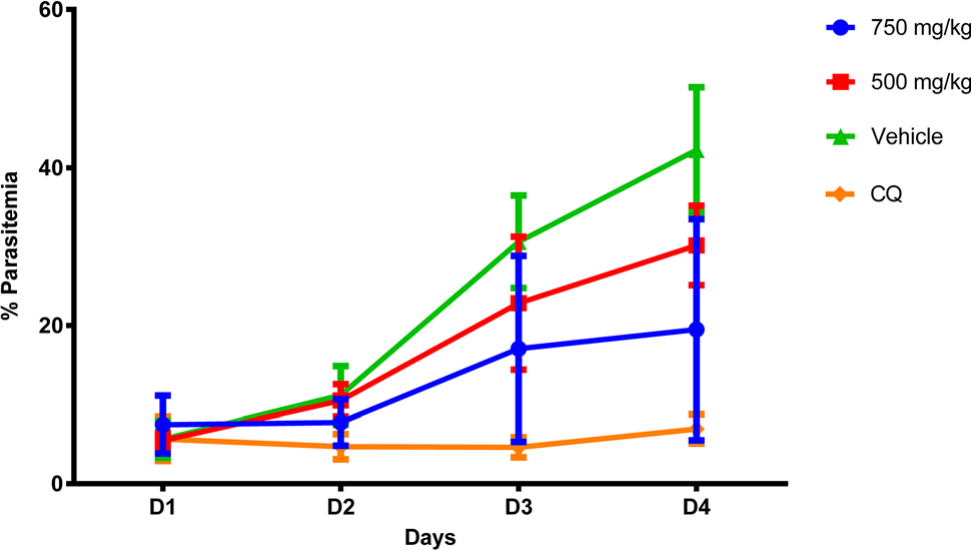
Prophylactic assay of *C. nucifera* extracts against *P. berghei*. Parasite growth was determined by flow cytometry and compared to culture controls with only vehicle added. Results shown are the mean of 5 mice ± SEM values

At the end of treatment, the parasitemia percentage in infected mice treated with 500 and 750 mg/kg/day was 30.16 ± 5.03 and 23.28 ± 12.89, respectively (Fig. 4). The untreated group had a percentage parasitemia of 42.25 ± 7.89, whereas that of the chloroquine-treated control was 6.95 ± 1.84. The percentage chemosuppression of parasites in mice treated with 500 and 750 mg/kg/day was 28.60% and 44.91%, whereas that of the treatment with chloroquine was 83.56%.

## Discussion

We carried out experiments to test the antimalarial activity of the extract from the leaves of *C. nucifera* on mice infected with *P. berghei* parasites, examining the routes of administration, the acute toxicity and different assays to determine its antiplasmodial effects.

The IM-treated mice were less susceptible to weight loss when compared with the other two routes of administration, oral and subcutaneous. Additionally, there was a greater antiplasmodial effect in these animals when compared to those treated subcutaneously and intragastrically. The latter administration of the extract involves the drug being subjected to the stomach acids which may cause it to be degraded (Levine 1970). Besides, using a gavage administration probably affected the animals’ ability to eat properly, resulting in weight loss, while subcutaneous administration may hinder the extract from reaching circulation and may not act against the parasites since the rate of absorption in this route is lower than intramuscular administration (Simmons and Brick 1970).

Our results for the acute toxicity assay showed that the extract presented signs of toxicity at the maximum concentration used (2000 mg/kg/day) when administered intramuscularly. In the group treated with the extract, two of the five mice died, with deaths beginning on the 6th day, suggesting that the dose at which half of the population dies (LD_50_) lies slightly beyond 2000 mg/kg/day, since no animals died at 1000 mg/kg/day. Blood analysis of hematocrit and hemoglobin levels in all groups remained generally within the normal values up until day 8, when the three concentrations used apparently caused the hemoglobin to fall below the lower normal limit. This trend continued until day 16th suggesting that the extract negatively affects the integrity of red blood cells if used for periods longer than 8 days. Further experiments should monitor these levels more closely to determine the effects on hematocrit and hemoglobin parameters of the mice on a daily basis. Additionally, mean cell volume (MCV) parameters were analyzed in all groups. MCV is a measure of the size of red blood cells and low values (normal reference range is typically 42.3–55.2 fl/red cell) are indicative of microcytic anemia (Dasgupta 2015).This parameter was found to be normal after inoculating the extract in all treated groups, indicating that, while the extract affects hemoglobin and hematocrit levels after the first 8 days, the size of the RBCs remains unaffected for extended periods of treatment.

At concentrations of 500 and 750 mg/kg/day, the leaf extract was able to exert a parasite chemosuppression of 51% and 54%, respectively. The slightly higher activity achieved at 750 mg/kg/day suggests that this extract has a moderate suppressive activity (Deharo et al. 2001). Tests using concentrations between 750 and 1000 mg/kg/day should be explored to improve chemosuppression, but avoiding the slight toxicity found in the mice at 1000 mg/kg/day.

The extract does not show any curative effect when tested at concentrations of 500 and 750 mg/kg/day on *P. berghei*—infected mice. Nonetheless, in the prophylactic test, the extract at 500 and 750 mg/kg/day showed a dose-dependent activity against the parasite with *P. falciparum* inhibitory percentages of 28.60% and 44.91%, respectively. For a comparison with a classic anti-malarial drug, the use of chloroquine before infection was 83.56% efficient in stopping the infection.

Different products from the coconut plant have been studied extensively for their medicinal properties (Roopan 2016). Research has demonstrated the presence of metabolites such as polyphenols, glycosides, steroids, alkaloids, terpenoids, and others which could be responsible for those activities (Renjith et al. 2013; Manalo et al. 2017; Oliveira et al. 2009; Singla et al. 2011; Soumya et al. 2014). More specifically, the husk extracts of *C. nucifera* have shown to have antimalarial properties when tested in vitro and in vivo (Adebayo et al. 2012; Adebayo et al. 2013; Balogun et al. 2014; Angeles et al. 2005).

Previous research by our group (Tayler et al. 2019) demonstrated the presence of flavan-3-ols (epicatechin), flavones (catechin derivatives, isoorientin, apigenin, vitexin, isovitexin, and luteolin) in the leaves of *C. nucifera* from Punta Patiño, Panama, and the in vitro antiplasmodial activity of a water decoction of these leaves was described.

Flavonoids are phenolic compounds and one of the most common secondary metabolites found in plants. Flavonoids such as acacetin, apigenin, baicalein, chrysin, genistein, kaempferol, luteolin, among many others have been identified to possess antimalarial activity (Lehane and Saliba 2008). Additionally, compounds from the catechin family have been involved in antimalarial action (Sannella et al. 2007).

Fatty acid synthesis is of utmost importance in the development of malarial parasites. They are needed for parasite membrane and lipid body biogenesis (Palacpac et al. 2004) and they are used for the anchorage of parasite membrane proteins through glycosylphosphatidylinositol moieties (Gilson et al. 2006). There are three important enzymes involved in the fatty acid biosynthesis of *P. falciparum*, namely 3-hydroxyacyl-[acyl-carrier-protein] dehydratase (FabZ), 3-oxoacyl-[acyl-carrier-protein] reductase (FabG), and Enoyl-[acyl-carrier-protein] reductase [NADH] (FabI) (Tasdemir et al. 2006). In a study from Tasdemir et al. (2006) it was demonstrated that the flavonoids belonging to the gallic acid esters of catechins were the most active compounds against the parasite. They performed kinetic analyses using luteolin and (-)-catechin gallate as model compounds and revealed that FabZ was inhibited competitively. FabG was inhibited in a noncompetitive manner, whereas both compounds behaved as tight-binding noncompetitive inhibitors of FabI. Additionally, these polyphenols showed in vitro activity against chloroquine-sensitive (NF54) and -resistant (K1) *P. falciparum* strains in the low to submicromolar range (Tasdemir et al. 2006). Furthermore, in the presence of catechins from green tea and other important plant polyphenols, the inhibitory effect of triclosan binding to FabI from *P. falciparum* was potentiated (Sharma et al. 2007). The heightened binding of triclosan because of the high affinity of catechins was furthermore explained by molecular modeling studies based on flavonoids luteolin, quercetin, fistein, (-)-catechin gallate, and others (Banerjee et al. 2008). Modelling also correlated with the activity of luteolin against chloroquine-sensitive and chloroquine-resistant strains of *P. falciparum*, following in silico studies where it was shown that luteolin had good binding affinity against dihydrooroate dehydrogenase (PfDHODH) and plasma membrane P-type cation translocating ATPase (PfATP4), two enzymes needed for the survival of the parasite. In vitro testing confirmed the finding showing low micromolar activity againt *P. falciparum*. The flavonoids were found in the juice of Citrus species used traditionally for the treatment of malaria-associated fevers (Gogoi et al. 2019).

Several studies describe how catechin-related compounds such as epigallocatechin-3-gallate (EGCG) and epicatechin gallate (ECG) show moderate antiplasmodial effects. In a study by Sannella et al. (2007), a crude extract, as well as EGCG and ECG from green tea leaves, were found to strongly inhibit the growth of *P. falciparum* parasites in vitro. Additionally, they found that these catechins were able to potentiate the effect of artemisinin when used in combination against malaria parasites (Sannella et al. 2007).

The presence of flavan-3-ols (epicatechin), flavones (catechin derivatives, isoorientin and luteolin, among many others) could help explain the parasitemia reduction of *P. berghei*-infected mice tested in this study. It is clear, from the chemosupression percentages of 51% and 54% in a 4-day suppressive assay, in addition to a moderate prophylactic actity, that our crude extract from *C. nucifera* possesses antimalarial properties. Acute toxicity assays, however, showed a slight harming effect of this extract on hematocrit and hemoglobin parameters for the group which showed 54% chemosuppression. Even with this finding, the extract from *C. nucifera* leaves is a candidate for further analysis using a bioassay-guided isolation of compounds and/or fractions selected from the whole decoction. In addition, it would be valuable to determine the combinatorial effect this extract, and/or its compounds, could have on malaria parasites when paired with standard drugs. These approaches could result in new candidates and/or structures for drug development against human malaria.
